# A geometric method for computing ocular kinematics and classifying gaze events using monocular remote eye tracking in a robotic environment

**DOI:** 10.1186/s12984-015-0107-4

**Published:** 2016-01-26

**Authors:** Tarkeshwar Singh, Christopher M. Perry, Troy M. Herter

**Affiliations:** Department of Exercise Science, Arnold School of Public Health, University of South Carolina, 921 Assembly Street, Columbia, SC-29208 USA

**Keywords:** Eye tracking, Upper limb, Robotics, Saccades, Fixations, Smooth pursuits, Eye-hand coordination

## Abstract

**Background:**

Robotic and virtual-reality systems offer tremendous potential for improving assessment and rehabilitation of neurological disorders affecting the upper extremity. A key feature of these systems is that visual stimuli are often presented within the same workspace as the hands (i.e., peripersonal space). Integrating video-based remote eye tracking with robotic and virtual-reality systems can provide an additional tool for investigating how cognitive processes influence visuomotor learning and rehabilitation of the upper extremity. However, remote eye tracking systems typically compute ocular kinematics by assuming eye movements are made in a plane with constant depth (e.g. frontal plane). When visual stimuli are presented at variable depths (e.g. transverse plane), eye movements have a vergence component that may influence reliable detection of gaze events (fixations, smooth pursuits and saccades). To our knowledge, there are no available methods to classify gaze events in the transverse plane for monocular remote eye tracking systems.

Here we present a geometrical method to compute ocular kinematics from a monocular remote eye tracking system when visual stimuli are presented in the transverse plane. We then use the obtained kinematics to compute velocity-based thresholds that allow us to accurately identify onsets and offsets of fixations, saccades and smooth pursuits. Finally, we validate our algorithm by comparing the gaze events computed by the algorithm with those obtained from the eye-tracking software and manual digitization.

**Results:**

Within the transverse plane, our algorithm reliably differentiates saccades from fixations (static visual stimuli) and smooth pursuits from saccades and fixations when visual stimuli are dynamic.

**Conclusions:**

The proposed methods provide advancements for examining eye movements in robotic and virtual-reality systems. Our methods can also be used with other video-based or tablet-based systems in which eye movements are performed in a peripersonal plane with variable depth.

**Electronic supplementary material:**

The online version of this article (doi:10.1186/s12984-015-0107-4) contains supplementary material, which is available to authorized users.

## Background

Commercial availability of robotic devices for neurological assessment and rehabilitation of the upper extremity has increased exponentially since 1998 [reviewed in 1, 2]. Assessment robots address a chronic need to obtain objective and quantitative measures of sensory, motor and cognitive function. Rehabilitation robots address a longstanding need to provide greater doses of challenging, adaptable and stimulating activities that can engage clinical populations for sustained periods of time [3]. Accordingly, over the last few years there has been a substantial increase in the volume of research combining virtual reality with upper limb robotics [reviewed in 3, 4].

A collateral benefit that has emerged from the development of robotic and virtual reality technologies is the potential to study how perception and cognition contribute to eye-hand coordination [5, 6]. This possibility can be realized by adding video-based eye tracking to upper-extremity robots. Video-based eye trackers can non-invasively obtain information on where a subject is directly looking, commonly referred to as the “point-of-regard (POR)” [7]. This information allows researchers to quantify overt mechanisms [8, 9] of visual search by ascertaining if objects of interest have been directly viewed (foveated).

Historically, visual stimuli in video-based systems have been presented on a computer monitor placed parallel to the frontal plane, and participants have responded by moving joysticks, a mouse, or other devices located within the peripersonal workspace [10, 11]. Presenting stimuli in the vertical plane ensures that the Euclidean distance of the stimuli from the eyes is relatively constant, which allows for relatively simple calculation of ocular kinematics and subsequent identification of onsets and offsets of gaze events (fixations, saccades, smooth pursuits). One criticism of this paradigm is that eye and hand movements are performed in two different workspaces, whereas most real-world eye-hand coordination tasks involve coordination of the eyes and hands in a common workspace. In contrast, the combination of robotic technology and virtual reality can replicate ecological conditions, such as reaching for a cup placed on a table [reviewed in 1, 3, 12, 13]. Recently, video-based eye tracking has been successfully integrated with robotic devices and virtual reality systems (e.g. KINARM Labs, The MotionMonitor), permitting studies of eye and hand movements in ecological conditions.

Although remote systems normally provide accurate gaze POR data in Cartesian coordinates, ocular kinematics are typically computed inversely from either the coordinates of intersection of the gaze vector with the stimulus plane (*gaze POR*) or with an arbitrary plane at an infinite distance (*gaze direction*). In both cases, the inverse computation (as specified in the user manuals) assumes that gaze events occur in a plane with constant depth and, consequently, gaze angle is computed as a scalar angle at each sampled time. This works reasonably well when depth of the stimulus plane is fixed during eye movements and there are minimal vergence eye movements [14, 15]. However, when stimuli are presented in peripersonal space, there are interactions between saccadic and vergence motor commands [16, 17]. To account for rotational features of eye movements that combine saccades and vergence [15], eye kinematics are better represented in a vector representation, such as Fick’s, Helmholtz’s or pitch-yaw-roll [18, 19]. Therefore, to obtain an accurate measure of ocular kinematics, its inverse computation from gaze POR should follow the mathematical rules that apply to transforming coordinates from Cartesian space to 3D spherical systems. Our first objective was to develop a method for computing ocular kinematics from gaze POR data in the transverse plane. The method presented here is based on the KINARM robotic device (BKIN Technologies, Kingston, ON, Canada) with integrated remote monocular eye tracking (SR EyeLink® 1000 Remote system). This method can be extended to other robotic and virtual reality systems.

A second issue we consider is the use of kinematic thresholds for distinguishing gaze-events. Standard position (0.15°) and velocity (30°/s) thresholds have their historical origins in the reading and scene perception literature [10, 20–22], and may not be valid within robotic environments for three reasons. First, visual stimuli in robotic experiments are typically large (0.5-1.5°) and spaced farther apart than words in reading studies. Since saccade amplitude and velocity are strongly correlated [23], standard thresholds used to separate gaze events may be too small in the robotic environment. Second, saccadic velocities may vary across individuals when studying patients whose oculomotor functioning may be altered to varying degrees [24, 25]. Third, vergence eye movements in the transverse plane would slow down saccade velocities, which may also influence eye movement thresholds. Therefore, our second objective was to propose a novel method for identifying thresholds that are task, environment, and participant specific.

Our third objective was to combine our algorithms for computing ocular kinematics and velocity thresholds to improve classification of gaze events (fixations, saccades, smooth pursuits). To validate our methods, we compare the classification accuracy of our methods (*Algorithm Classification*) with gaze events computed using two other methods: a) gaze event classification by the eye-tracker (*SR Classification*); and b) manual digitization of the eye events by marking positional traces of gaze POR (*Manual Classification)*.

## Methods

### Apparatus

**Fig. 1:**
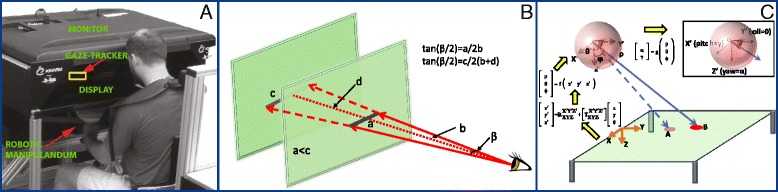
The experimental setup and theoretical paradigm used to compute ocular kinematics for the current study. **a** The KINARM Endpoint robot with a seated participant. The visual stimuli are reflected from the monitor to the display. The remote gaze-tracker is housed at the back of the workspace (yellow rectangle). Participants rest their head against a support in front of the monitor and grab the robotic manipulanda just below the display to interact with the visual stimuli. **b** Along the line of sight, visual angle (β) spans diameters ‘a’ and ‘c’ at two different distances. In psychophysics studies, visual stimuli are often presented in a frontal plane and converted to degrees to eliminate distance as a confounding variable. **c** A cartoon of an arbitrary gaze point-of-regard (POR) first transformed from the robot based (XY) to an eye-based (X’Y’Z’) Cartesian coordinate system. Note that the Z-axis (pointing downward, robot frame) is shown to illustrate how Eq. 2 transforms the gaze POR data. This is followed by a transformation to a spherical coordinate system, also fixed to the eye. Then ocular kinematics are obtained from the spherical gaze POR data (see inset) using Equations 6–8. The yellow arrows indicate the sequence in which this transformation is performed

We used a KINARM Endpoint robot (BKIN Technologies, Kingston, ON, Canada) with an integrated EyeLink 1000 Remote eye tracking system (SR Research, Ottawa, ON, Canada) that is mounted about 80 cm in front of the participant’s eyes (Fig. 1a). Participants grasp handles to interact with targets that are projected onto the same plane as the hands using a monitor and semi-transparent mirror mounted above the workspace [26]. The eye tracking system is a monocular system with a maximum sampling frequency of 500 Hz, accuracy of 0.5° and microsaccade resolution of 0.25°. Head position is measured using a target placed on the participant's forehead and the pupils are detected with a proprietary algorithm. The eye tracker is calibrated for the 2D horizontal workspace using proprietary algorithms (BKIN Technologies). The eye tracking output from the robot consists of X and Y position data (gaze POR) in the stimulus plane, timestamps, pupil size, and onset and offset of fixations and saccades. Except for the gaze POR data, all data are passed directly (without processing) from the EyeLink system to the KINARM system.

### Summary of gaze data preprocessing, transformations and classification

First, we preprocess data by removing blinks and gaze artifacts. After blinks and artifacts are removed, all the data are low pass filtered at 20 Hz before further processing. Second, we transform gaze POR data from 2D Cartesian coordinates on the robotic display in the transverse plane to an eye-based 3D spherical coordinate system. Third, we use the derivatives of the spherical coordinates to inverse compute gaze kinematics (eye-in-head yaw, pitch and roll). Then we use a novel method based on the statistical distribution of gaze velocity peaks obtained from the kinematics to compute velocity thresholds for classifying gaze events. Finally, we combine ocular kinematics, velocity thresholds and dynamics of the visual stimuli to propose a novel algorithm for classifying gaze events.

### Blinks and artifacts removal steps

Our preprocessing steps are almost identical to those proposed by Larsson and colleagues [27]. We first take care of blinks (closed eyelids), one sample spikes (incorrect detection of corneal reflection) and screen outliers (instances when gaze wanders outside the workspace). If the eyelid closes or the gaze-tracker fails to detect the pupil, it assigns a special value (e.g., −100) to the x and y gaze POR coordinates. By detecting these values in the raw gaze positions signals, blinks are eliminated by performing a cubic spline interpolation between the last five and first five points before and after blink onset and termination. The same rule is applied for screen outliers. Finally, one sample spikes are removed by replacing them with the neighboring samples that are closest in value [28].

### Transforming gaze POR data from a 2D plane to an eye-based 3D spherical coordinate system

For remote eye tracking systems, only the gaze POR information is typically provided and ocular kinematics are inversely computed from the gaze POR or eye in head coordinates (gaze direction). Irrespective of the particular measure, ocular kinematics are commonly computed under the assumption that gaze is restricted to a frontal plane (cf. SR EyeLink and Tobii user manuals). Under this assumption, computing the angular kinematics involves a simple conversion of Cartesian data to angular form. Specifically, a scalar visual angle (β) is calculated assuming distance ‘b’ is fixed along the line of sight (see Fig. 1b) and is perpendicular to the viewing plane [14, 29]. Equation 1 gives the relationship between viewing distance ‘b’, visual angle β, and distance on the display, ‘a’:

1 $$ \tan \left(\frac{\upbeta}{2}\right)=\frac{\mathrm{a}}{2\mathrm{b}} $$

A scalar form of ‘β’ is a simplification of ocular kinematics and it works reasonably well when ‘b’ is fixed during an eye movement. However, when ‘b’ is not fixed and eye movements also have a depth component [15, 17], such as within a transverse peripersonal space, a vector form of the ocular kinematics should be computed.

We first transform the 2D Cartesian coordinates of gaze POR in the transverse plane to a 3D eye-based spherical coordinate system. We assume that the output from the eye tracking system at every sampled instant is in the (x, y) format, where x and y are the Cartesian coordinates of the gaze POR in the transverse plane with positive x-axis going away from the participant in the right lateral direction and positive y-axis going away from the participant in the anterior direction (Fig. 1c). We then transform gaze data from this 2D Cartesian system (XY) to a 3D eye-based Cartesian coordinate system (X’Y’Z’) that is assumed to be fixed and centered at the center of the spherical eye. This transformation assumes that the head is relatively stationary and the height (H) of the eye from the stimulus plane is known and fixed. Equation 2 transforms the 2D gaze POR to the 3D eye-based Cartesian coordinate system:

2 $$ \left[\begin{array}{c}\hfill \mathrm{x}\mathit{\hbox{'}}\hfill \\ {}\hfill \mathrm{y}\mathit{\hbox{'}}\hfill \\ {}\hfill \mathrm{z}\mathit{\hbox{'}}\hfill \end{array}\right]=\left[\begin{array}{c}\hfill 0\hfill \\ {}\hfill 0\hfill \\ {}\hfill \mathrm{H}\hfill \end{array}\right]+\left[{\mathrm{R}}_{\mathrm{x}\mathrm{y}\mathrm{z}}^{\mathrm{x}\mathit{\hbox{'}}\mathrm{y}\mathit{\hbox{'}}\mathrm{z}\mathit{\hbox{'}}}\right]\left[\begin{array}{c}\hfill \mathrm{x}\hfill \\ {}\hfill \mathrm{y}\hfill \\ {}\hfill 0\hfill \end{array}\right] $$

The ‘0’ in the last vector on the right hand side of Equation 2 implies that the gaze POR is in the horizontal XY plane. The orientation matrix in Equation 2, [R_xyz_^x ' y ' z '^], prescribes the relative tilt of the head with respect to the robot based coordinate frame. Gaze data in the eye-based Cartesian system (x’, y’, z’) is then transformed to an eye-based spherical coordinate system (ρ, θ, ϕ). It is assumed that the origin of the X’Y’Z’ and spherical coordinate systems coincide. ‘ρ’ is the radial distance of the gaze POR on the viewing plane from the origin of the spherical coordinate system. ‘θ’ is the azimuthal angle in the X’Y’-plane from the X’-axis with 0 ≤ θ <2π. ‘ϕ’ is the elevation angle and is measured from the positive Z’-axis with 0 ≤ ϕ ≤ π. The Cartesian to spherical transformation is non-linear (shown in Fig. 1c as (ρ, θ, ϕ) = f (x’, y’, z’)) and is performed using Equation 3:

3a $$ \uprho =\sqrt{\mathrm{x}{\mathit{\hbox{'}}}^2+\mathrm{y}{\mathit{\hbox{'}}}^2+\mathrm{z}{\mathit{\hbox{'}}}^2} $$

3b $$ \uptheta ={ \tan}^{-1}\left(\frac{\mathrm{y}\mathit{\hbox{'}}}{\mathrm{x}\mathit{\hbox{'}}}\right) $$

3c $$ \upphi ={ \cos}^{-1}\left(\frac{\mathrm{z}\mathit{\hbox{'}}}{\uprho}\right) $$

### Computing gaze angular velocity from the 3D Cartesian POR data

Differentiating ϕ and θ yields $\overset{.}{\upphi}$ and $\overset{.}{\uptheta}$, respectively, as described in Equation 4:

4a $$ \overset{.}{\upphi} = \frac{\mathrm{z}\mathit{\hbox{'}}\left(\mathrm{x}\mathit{\hbox{'}}\overset{.}{\mathrm{x}}\mathit{\hbox{'}}+\mathrm{y}\mathit{\hbox{'}}\overset{.}{\mathrm{y}}\mathit{\hbox{'}}\right)-\left(\mathrm{x}{\mathit{\hbox{'}}}^2+\mathrm{y}{\mathit{\hbox{'}}}^2\right)\overset{.}{\mathrm{z}}\mathit{\hbox{'}}}{\left(\mathrm{x}{\mathit{\hbox{'}}}^2+\mathrm{y}{\mathit{\hbox{'}}}^2+\mathrm{z}{\mathit{\hbox{'}}}^2\right)\sqrt{\left(\mathrm{x}{\mathit{\hbox{'}}}^2+\mathrm{y}{\mathit{\hbox{'}}}^2\right)}} $$

4b $$ \overset{.}{\uptheta}=\frac{\overset{.}{\mathrm{x}}\mathit{\hbox{'}}\mathrm{y}\mathit{\hbox{'}}-\mathrm{x}\mathit{\hbox{'}}\overset{.}{\mathrm{y}}\mathit{\hbox{'}}}{\left(\mathrm{x}{\mathit{\hbox{'}}}^2+\mathrm{y}{\mathit{\hbox{'}}}^2\right)} $$

To compute $\overset{.}{\upphi}$ and $\overset{.}{\uptheta}$, gaze POR velocity in Cartesian coordinates ($\overset{.}{\mathrm{x}}\mathit{\hbox{'}},\ \overset{.}{\mathrm{y}}\mathit{\hbox{'}}\mathrm{and}\ \overset{.}{\mathrm{z}}\mathit{\hbox{'}}$) is first calculated using Savitzky-Golay differentiation and smoothing [30]. Equation 5 gives the velocity vector in spherical coordinates:

5 $$ \mathrm{v}=\overset{.}{\uprho}\widehat{\uprho}+\uprho \overset{.}{\uptheta} \sin \left(\upphi \right)\widehat{\uptheta}+\uprho \overset{.}{\upphi}\widehat{\upphi} $$

Here $\left(\widehat{\uprho},\kern0.5em \widehat{\uptheta}\kern0.75em \mathrm{and}\kern0.5em \widehat{\upphi}\right)$ denote the unit vector in those directions. Since the spherical basis vectors are orthonormal, Equations 6a and b represent the magnitudes of the angular velocity and acceleration respectively:

6a $$ {\mathrm{v}}_{\parallel \upphi, \kern0.5em \uptheta \parallel }=\sqrt{{\left(\overset{.}{\uptheta} \sin \left(\upphi \right)\right)}^2+{\left(\overset{.}{\upphi}\right)}^2} $$

6b $$ {\overset{.}{\mathrm{v}}}_{\parallel \upphi, \kern0.5em \uptheta \parallel }=\sqrt{{\left(\frac{2\overset{.}{\uprho}\overset{.}{\uptheta} \sin \upphi}{\uprho}+\ddot{\uptheta} \sin \upphi -2\overset{.}{\uptheta}\overset{.}{\upphi} \cos \upphi \right)}^2+{\left(\frac{2\overset{.}{\uprho}\overset{.}{\upphi}}{\uprho}+{\overset{.}{\upphi}}^2 \sin \upphi \cos \upphi +\ddot{\upphi}\right)}^2} $$

### Transforming spherical coordinates to oculomotor kinematics

The spherical coordinates are mapped to angular rotations of the eye (inset, Fig. 1c) with the following empirical steps: A translation of a point at the origin by a distance ρ along the Z’ axis; followed by a rotation (ϕ) about the X’ axis; and finally a (90-θ) rotation about the Z’ axis [31]. The rotational component of this transformation is then given as a product of elementary rotations about the X’ and Z’ axes, as described in Equation 7:

7 $$ {\mathrm{R}}_{\mathrm{Z}\mathit{\hbox{'}},\kern0.5em 90-\uptheta}{\mathrm{R}}_{\mathrm{X}\mathit{\hbox{'}},\kern0.5em \upphi}=\left[\begin{array}{ccc}\hfill \sin \left(\uptheta \right)\hfill & \hfill - \cos \left(\upphi \right) \cos \left(\uptheta \right)\hfill & \hfill \sin \left(\upphi \right) \cos \left(\uptheta \right)\hfill \\ {}\hfill \cos \left(\uptheta \right)\hfill & \hfill \cos \left(\upphi \right) \sin \left(\uptheta \right)\hfill & \hfill - \sin \left(\upphi \right) \sin \left(\uptheta \right)\hfill \\ {}\hfill 0\hfill & \hfill \sin \left(\upphi \right)\hfill & \hfill \cos \left(\upphi \right)\hfill \end{array}\right] $$

Figure 1c demonstrates how the rotation matrix in Equation 7 rotates the gaze vector from position OA to OB.

To map spherical coordinates to ocular kinematics, we assume that Listing’s law is neurally implemented with a two-dimensional input signal (to the oculomotor plant) that is confined to a pitch-yaw plane (see inset of Fig. 1c) [10, 32–34]. In other words, pitch and yaw angles uniquely prescribe the roll angle. Furthermore, we assume that the axes are fixed to the head. Thus, as long as the head is relatively restrained, the yaw-pitch-roll axes are fixed in space. Equation 8 gives the rotation matrix for pitch and yaw rotations:

8 $$ {\mathrm{R}}_{\mathrm{Z},\upgamma}{\mathrm{R}}_{\mathrm{X},\upalpha}=\left[\begin{array}{ccc}\hfill \cos \left(\upgamma \right)\hfill & \hfill - \cos \left(\upalpha \right) \sin \left(\upgamma \right)\hfill & \hfill \sin \left(\upalpha \right) \sin \left(\upgamma \right)\hfill \\ {}\hfill \sin \left(\upgamma \right)\hfill & \hfill \cos \left(\upalpha \right) \cos \left(\upgamma \right)\hfill & \hfill - \sin \left(\upalpha \right) \cos \left(\upgamma \right)\hfill \\ {}\hfill 0\hfill & \hfill \sin \left(\upalpha \right)\hfill & \hfill \cos \left(\upalpha \right)\hfill \end{array}\right] $$

On comparing the rotation matrices in Eq. 7 and 8, it is evident that α = ϕ and γ = 90-θ. Therefore, we substitute the spherical coordinates ($\upphi,\ \uptheta, \kern0.5em \overset{.}{\upphi},\kern0.5em \overset{.}{\uptheta}$) for the ocular kinematics $\left(\upalpha, \kern0.5em \upgamma, \kern0.5em \overset{.}{\upalpha},\kern0.5em \overset{.}{\upgamma}\right)$ and use those to quantify gaze events (saccades, smooth pursuits, fixations).

### Computing the foveal visual radius

**Fig. 2:**
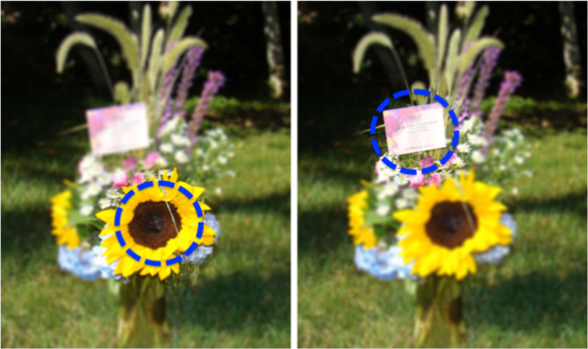
A flower bouquet and an attached greeting card viewed from a distance of ~1 m demonstrates the challenge associated with the drop in visual acuity outside the foveal region. At any given instance the bouquet can only be foveated partially. **a** When the flower is foveated, the details on the card cannot be read. **b** When the greeting card is foveated, the details of the flower are obscured

The fovea, which covers about 2-3° of visual angle from the center of the gaze, is specialized for maximum visual acuity, which drops steeply outside the fovea. Acuity is reduced by almost 75 % at 6° eccentricity [35] and by 90 % at 20° eccentricity [36]. This makes it difficult to ascertain the focus of foveal vision when a visual scene has multiple objects (e.g. the flower and greeting card in Fig. 2 cannot be foveated simultaneously). To solve this problem, we compute the Foveal Visual Circle (FVC), which assumes that visual acuity is high within the FVC and low outside the FVC. The center of the FVC is assumed to be located at the gaze POR (x’, y’, z’). The radius of the circle, FVR, is then computed using Equation 9:

9 $$ \mathrm{F}\mathrm{V}\mathrm{R}\approx \uprho \left( \tan \left(\frac{\updelta}{2}\right)\right)\mathrm{cosec}\left(\upvarepsilon \right) $$

**Fig. 3:**
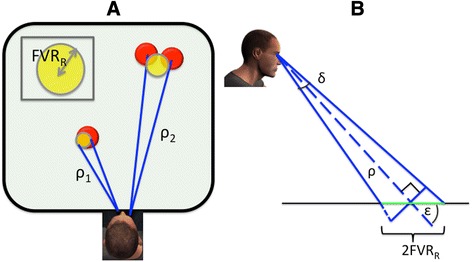
The visual foveal radius increases as a function of ρ, which implies that, at larger distances, participants can foveate and discriminate multiple objects simultaneously. **a** Since ρ_2_ > ρ_1_, the FVR (also see inset) for ρ_2_ is larger. If the participant places the center of the foveal circle strategically during visual search, multiple objects (red circles) could be foveated simultaneously. **b** Schematic diagram of how the FVR is computed from the eye tracking data

Here, ‘δ’ is the visual angle (taken as 3° + mean calibration error obtained from the calibration procedure for an experimental session), ‘ρ’ is the magnitude of the gaze radius vector from Equation 3 and ‘ε’ is the angle between the gaze radius vector and the stimulus plane. FVR is distance dependent, it is larger when gaze is fixated or pursuing an object farther away in peripersonal space (see Fig. 3a). A schematic for the computation of FVR is shown in Fig. 3b. We assume that a target is overtly foveated if any of its edges overlap with the FVC, though this is a simplistic model of foveal visual acuity. In reality, cone density decreases gradually at the periphery of the fovea. However, modeling these continuous changes is outside the scope of this paper.

### Classification of fixation and saccades

To differentiate saccades and fixations, velocity and acceleration thresholds of 30°/s and 6000°/s^2^ have been proposed in the literature [10, 11, 20–22]. These thresholds have been derived primarily from the reading and scene perception literature, and may not directly apply to hand-eye coordination studies in robotic environments where stimuli are larger and usually spaced farther apart than words. We implemented a novel method to obtain velocity-based thresholds from the local gaze velocity peaks.

We extracted the local velocity peaks from the time series of gaze velocity (v_∥ϕ, θ∥_) and used the maximum likelihood method to fit a bimodal lognormal distribution to the vector of velocity peaks (see Figure and MATLAB function in Additional files 1 and 2 in Supplementary Materials). If a task primarily involves only fixations and saccades, the first mode captures local velocity peaks due to noise and microsaccades made during fixations [37] and the second mode captures the local velocity peaks during saccades. The parameters of the two distributions are then used to create a decision variable [38]:

10a $$ \mathrm{Saccade}\ \mathrm{Velocity}\ \mathrm{Thresold} = {\mathrm{v}}_{\parallel \upphi, \kern0.5em \uptheta \parallel}^{\mathrm{Thres},\kern0.5em \mathrm{Saccade}}= \exp \left[0.5\times \left\{\left({\upmu}_2-2{\upsigma}_2\right)+\left({\upmu}_1+2{\upsigma}_1\right)\right\}\right] $$

10b $$ \mathrm{Saccade}\ \mathrm{Acceleration}\ \mathrm{Threshold}={\overset{.}{\mathrm{v}}}_{\parallel \upphi, \kern0.5em \uptheta \parallel}^{\mathrm{Thres},\ \mathrm{Saccade}}=6000{}^{\circ}/{\mathrm{s}}^2 $$

Here, ‘μ’ and ‘σ’ in Eq. 10a are the means and standard deviations of the first and second lognormal distributions. For each velocity peak that exceeds the Saccade Velocity Threshold, we confirm that the peak acceleration also exceeds the *Saccade Acceleration Threshold* (Equation 10b) and the duration of saccade exceeds the *Minimum Saccade Duration* (5 ms). If these two conditions are satisfied*,* we classify the gaze event as a saccade.

Once a gaze event is classified as a saccade, we determine the onset and offset by searching for the first inflection point before and after the local peak in v_∥ϕ,θ∥_. For fixations, gaze velocity should be lower than both the velocity and acceleration thresholds described in Equation 10. In addition, the following condition should be continuously satisfied for a *Minimum Fixation Duration* of at least 40 milliseconds [21] .

11 $$ \parallel \mathrm{G}\left(\mathrm{t}\right)-\mathrm{T}\left(\mathrm{t}\right)\parallel \kern0.5em \le \kern0.5em \left[\mathrm{F}\mathrm{V}\mathrm{R}\left(\mathrm{t}\right)+{\mathrm{T}}_{\mathrm{R}}\left(\mathrm{t}\right)\right] $$

Here, G is the gaze POR and T is the static position of the visual stimuli, both in Cartesian coordinates in the (X’Y’Z’) frame of reference. FVR is the foveal visual radius (Equation 9) and T_R_ is the radius of the smallest circle that circumscribes the shape of the visual stimuli.

### Classification of smooth pursuits, saccades and fixations

Here we present a generic algorithm for classifying smooth-pursuits, saccades and fixations for tasks that present multiple moving objects simultaneously. We use a combination of Cartesian and spherical coordinates to improve the reliability and accuracy of our algorithm. In addition to the Minimum Saccade Duration (5 ms) and Minimum Fixation Duration (40 ms) described above, the *Minimum Pursuit Duration* was set at 40 ms, corresponding to the shortest timespan for stimulus processing [39]. The following algorithm classifies smooth-pursuits, saccades and fixations. We assume that fixation events are rare if all stimuli are moving. Thus, we classify smooth pursuits first, then saccades and finally fixations.

**Fig. 4:**
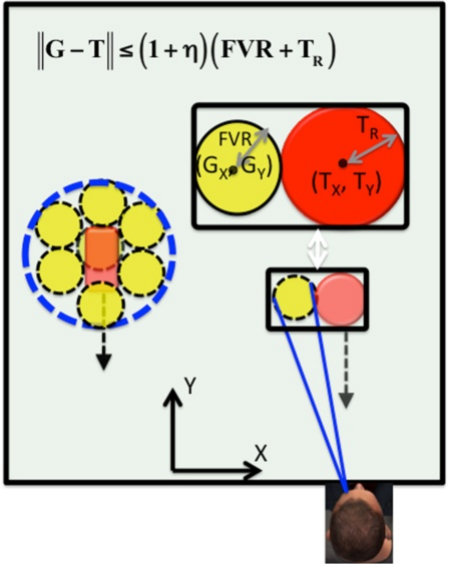
Representative example of smooth pursuits of objects moving in the horizontal plane towards the participant (negative Y direction). On the left is a vertical rectangle and the yellow circles show the locations of the foveal visual circles (FVC) that will result in a successful pursuit. The dashed blue ellipse shows the approximate area around the rectangle within which the FVCs need to lie for a successful pursuit. On the right is a FVC next to a circular red target. The inset shows an enlarged version of the circular target and FVC. For a successful pursuit, the distance between the center of the FVC and target should be continuously less than or equal to the sum of the foveal visual radius (FVR) and target radius for at least the *Minimum Pursuit Duration* (40 ms). The equation in the top part of the Figure shows the conditions that ought to be continuously satisfied for the *Minimum Pursuit Duration*

Equations 12a-d compare positions and velocities of the gaze and target and also check if gaze velocities are below the saccade velocity threshold obtained from Equation 10a. Because pursuit of targets with predictable movement are driven by an internal estimate of target speed (rather than retinal slip only), smooth pursuits can also lead objects [reviewed in 40]. Therefore, our algorithm considers that gaze can either lag or lead targets but by not more than the FVR (also see Fig. 4).

12a $$ \parallel \mathrm{G}\left(\mathrm{t}\right)\kern0.5em -\kern0.5em \mathrm{T}\left(\mathrm{t}\right)\parallel \kern0.5em \le \kern0.5em \left(1+\upeta \right)\left(\mathrm{F}\mathrm{V}\mathrm{R}\left(\mathrm{t}\right)+{\mathrm{T}}_{\mathrm{R}}\left(\mathrm{t}\right)\right) $$

12b $$ \parallel {\mathrm{v}}_{\parallel \upphi, \kern0.5em \uptheta \parallel }-{\overset{.}{\mathrm{T}}}_{\parallel \upphi, \kern0.5em \uptheta \parallel}\parallel \kern0.5em \le \kern0.5em \upeta \left({\overset{.}{\mathrm{T}}}_{\parallel \upphi, \kern0.5em \uptheta \parallel}\right) $$

12c $$ {\mathrm{v}}_{\parallel \upphi, \kern0.5em \uptheta \parallel }<{\mathrm{v}}_{\parallel \upphi, \kern0.5em \uptheta \parallel}^{\mathrm{Thres},\mathrm{Saccade}} $$

12d $$ {\overset{.}{\mathrm{v}}}_{\parallel \upphi, \kern0.5em \uptheta \parallel }<{\overset{.}{\mathrm{v}}}_{\parallel \upphi, \kern0.5em \uptheta \parallel}^{\mathrm{Thres},\ \mathrm{Saccade}} $$

Here, G is the gaze POR and T is the object position in Cartesian coordinates in the XYZ frame of reference. FVR is the foveal visual radius (Equation 9) and T_R_ is the radius of the smallest circle that circumscribes the object shape. v_∥ϕ, θ∥_ and ${\overset{.}{\mathrm{T}}}_{\parallel \upphi, \kern0.5em \uptheta \parallel }$ are the gaze and target angular velocities with respect to the eye-based spherical coordinate system (see Equation 6). The noise tolerance coefficient, ‘η’, is a correction factor to mitigate the effects of noise by expanding the field of high visual acuity and creating a larger tolerance for noise-induced fluctuations in gaze angular velocity. Finally, the gaze velocity and acceleration (leading up to the velocity peak) should be below the saccade thresholds.

Saccade thresholds are obtained using the same process as Equation 10a. However, when smooth pursuits are present, the threshold for distinguishing saccades from smooth pursuits and fixations would be higher compared to tasks with only saccades and fixations. In the presence of smooth pursuits, the first mode captures local velocity peaks due to noise, microsaccades and smooth pursuits, and the second mode captures velocity peaks of saccades. Smooth pursuit duration is recorded as the consecutive time points when all conditions in Equation 12 are satisfied. Pursuit duration must be greater than or equal to the Minimum Pursuit Duration of 40 ms.

Since smooth pursuit onset usually lags target appearance by about 100 ms, saccade termination at a target can be accompanied by a short fixation. Therefore, we look for fixations within 100 ms of saccade termination if this time epoch has not already been classified as a smooth pursuit. In addition to Equations 12c and 12d, the conditions in Equation 13 need to be satisfied. Equations 13a and 13b imply that the differences in angular position and velocity of the target and gaze are increasing (i.e., gaze is relatively stationary). Equation 13c states that the convex hull encompassing the gaze points during fixation overlaps the FVC of the gaze POR at the start of the fixation.

13a $$ {\overset{.}{\mathrm{T}}}_{\parallel \upphi, \kern0.5em \uptheta \parallel }>\upeta \times {\mathrm{v}}_{\parallel \upphi, \kern0.5em \uptheta \parallel } $$

13b $$ {\ddot{\mathrm{T}}}_{\parallel \upphi, \kern0.5em \uptheta \parallel }>\upeta \times {\overset{.}{\mathrm{v}}}_{\parallel \upphi, \kern0.5em \uptheta \parallel } $$

13c $$ \mathrm{convex}\ \mathrm{hull}\ \mathrm{area}\left({{\mathrm{G}}_{\mathrm{X}}}_{\left({\mathrm{t}}_{\mathrm{end}\_\mathrm{fixation}}-{\mathrm{t}}_{\mathrm{start}\_\mathrm{fixation}}\right)},{{\mathrm{G}}_{\mathrm{Y}}}_{\left({\mathrm{t}}_{\mathrm{end}\_\mathrm{fixation}}-{\mathrm{t}}_{\mathrm{start}\_\mathrm{fixation}}\right)}\right)\le \upeta \times \left(\uppi \times {\left({\mathrm{FVR}}_{\left({\mathrm{t}}_{\mathrm{start}\_\mathrm{fixation}}\right)}\right)}^2\right) $$

Here, FVR is centered at (${{\mathrm{G}}_{\mathrm{X}}}_{\left({\mathrm{t}}_{\mathrm{start}\_\mathrm{fixation}}\right)},\ {{\mathrm{G}}_{\mathrm{Y}}}_{\left({\mathrm{t}}_{\mathrm{start}\_\mathrm{fixation}}\right)}\Big)$. The start fixation time (t_start-fixation_) is the first time instant after saccade termination. The end fixation time (t_end-fixation_) is the first time instant when either of the conditions in Equations 13a and 13b is not satisfied.

### Experimental tasks

Three experiments were conducted as part of the study. Experiment One was used to compare how *Algorithm Classification* (classification by our algorithm) and *SR Classification* (SR EyeLink settings: 0.3°, 30°/s and 8000°/s^2^ thresholds for angular displacement, velocity and acceleration) fared against *Manual Classification*. For *Manual Classification,* an expert marked onsets and offsets of gaze events using gaze POR positional traces in Dexterit-E Explorer 1.2 (BKIN Technologies). Gaze events from *Algorithm Classification* and *Manual Classification* consisted of fixations, smooth pursuits and saccades. *SR Classification* gaze events included only saccades and fixations (limitation of the EyeLink system). A video of all three experiments is provided in the Supplementary Materials (Additional file 3).

In Experiments One and Two, the KINARM robot sampled data at 1000 Hz and the SR EyeLink system collected data at 500 Hz. The EyeLink data were upsampled offline before post-processing. In Experiment Three, both the KINARM robot and the SR EyeLink system sampled data at 200 Hz.

#### Experiment one

**Fig. 5:**
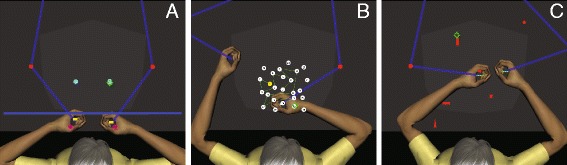
The three experimental tasks used in the current study. **a** This Figure shows an Experiment One trial in progress. This task was a bimanual task in which participants were instructed to hit targets (circles) and avoid hitting distractors (ellipses). The green cross hair indicates gaze POR. **b** The workspace for the Trail Making Test (Experiment Two). TMT is a unimanual task in which the goal was to reach all the numbers/letters in the correct order in the shortest possible time. The trial shown is a TMT-A trial and the participant has successfully traced numbers 1–16. **c** The workspace for the *Object Hit* and *Avoid* task (Experiment Three) is shown. This was a bimanual task in which participants used two virtual paddles to intercept and hit targets that are approaching them. For this trial, circles and vertical rectangles are the target shapes and all other shapes are distractors. At this moment case, the subject is about to hit the circle and is already looking ahead at the vertical rectangle

Experiment One involved a discrete bimanual task in which participants were asked to hit targets (using virtual paddles) and avoid hitting distractors. At the start of each trial, subjects moved both hands into two starting blocks (circles, 1 cm radius) located 12 cm in front of their body. A fixation circle then appeared 50 cm in front of the participants, who fixated the circle for a random interval of 500 to 1000 ms. The fixation circle then disappeared and two objects appeared on either side of the fixation circle’s location and moved towards the participants at the same speed, which varied randomly from 24 to 42 cm/s (see Fig. 5a). Either of the two objects could be a target (circle, 1 cm radius) or distractor (ellipse, 1.25 cm major and 1 cm minor axis radius). To perform the task, participants had to redirect their gaze to foveate both the moving objects, decide whether an object was a target or distractor, and make hand movements needed to hit the target(s). Participants were instructed to hit the targets before they passed a blue line located 18 cm in front of them. Objects stopped moving as soon as they were hit. Each experimental session consisted of 32 trials and each trial lasted about 1.5-3.5 s.

#### Experiment two

In this experiment, the Trail Making Tests (TMT) were used to classify saccades and fixations [41]. TMT are neuropsychological tests, similar to “connect-the-dots”, in which patients draw lines to connect numbers (TMT-A: 1, 2, 3…), or numbers and letters (TMT-B: 1, A, 2, B, 3, C…). Participants searched the numbers/letters by making saccades to them and fixating on them briefly before making a saccade to the next target (Fig. 5b). The task goal was to make reaching movements to all numbers and letters in the shortest possible time.

#### Experiment three

Experiment Three was used to classify smooth pursuits, saccades and fixations. In the Object Hit and Avoid (OHA) task, subjects used virtual paddles to interact with objects (300 objects in 8 geometric shapes) that moved towards the participants from the top of the workspace (see Fig. 5c). Participants were required to hit away 200 Targets (2 object shapes) and avoid hitting 100 Distractors (6 object shapes). The number of objects in the workspace and their velocity increased linearly with time. To discriminate targets and distractors, participants rapidly switched between smooth pursuits and saccades to foveate as many objects as possible. Each trial lasted about 140 s.

### Participants

Two participants volunteered to participate in Experiments One and Three. Sixty-seven participants including 37 young controls, 16 older controls (51–80 years) and 14 stroke survivors (*n* = 14, 48–80 years) volunteered to participate in Experiment Two. The Institutional Review Board of the University of South Carolina approved the study and all participants provided informed consent before beginning the experiment.

### Indices for computing classification accuracy

To quantify the classification rate, we used the data from Experiment One and computed indices that measured the proportion of data that were correctly classified by the algorithm [42]. In each trial, we manually identified the time points when participants initiated and terminated fixations, smooth pursuits and saccades. For example, let us assume, that in a trial with 3000 sampled time points, a fixation was manually classified between time points 201 and 800 as fixation (*Manual Classified fixation)* and our algorithm classified time points 191 to 750 as fixation (*Algorithm Classified fixation)*. A counter, *Manual Fixation Counter,* would start at time point 201 and increment in steps of 1 to 800 (i.e. final *Manual Fixation Counter* = 600). We then found the overlapping points between *Manual Classified fixation* and *Algorithm Classified fixation.* Thus, the *Algorithm Fixation Counter* would begin at 201 and increment to 750 in steps of 1 (i.e. *Algorithm Fixation Counter* = 550). This implies that our algorithm correctly classified a total of 550/600 points for this trial. We then computed a Fixation Quantitative Score (FQnS) as follows:

14 $$ \mathrm{FQnS}=\raisebox{1ex}{${\displaystyle {\sum}_{i=1}^{64}}100\times \frac{Algorithm\  Fixation\  Counte{r}_i}{Manual\  Fixation\  Counte{r}_i}$}\!\left/ \!\raisebox{-1ex}{$64$}\right. $$

Here, 64 in the summation sign and denominator indicate the number of trials (32 trials for each of the two participants) in Experiment One. Perfect classification by the algorithm would result in an FQnS score of 100 and if none of the points were correctly classified then FQnS will be equal to 0. In the example stated above, *Algorithm Fixation Counter*_*i*_*/Manual Fixation Counter*_*i*_ = 0.92 (550/600). A Saccade Quantitative Score (SQnS) and a Smooth Pursuit Quantitative Score (SPQnS) were also calculated exactly in the same fashion. The same process was repeated for comparing gaze events *from SR Classification* and *Manual Classification.*

We also computed indices for misclassified points. In the example, if the algorithm classified points 771 to 800 as smooth pursuits, 30 points would be misclassified. An index of misclassification of fixations (misFQnS) was computed as follows:

15 $$ \mathrm{misFQnS}=\raisebox{1ex}{${\displaystyle {\sum}_{i=1}^{64}}100\times \frac{Algorithm \sim Fixation\  Counte{r}_i}{Manual\  Fixation\  Counte{r}_i}$}\!\left/ \!\raisebox{-1ex}{$64$}\right. $$

Here, *Algorithm ~ Fixation Counter* indicates points that were classified as either saccades or smooth pursuits by the algorithm but as fixations manually. The same process was repeated for saccades and smooth pursuits.

The accuracy of *Algorithm Classification* depends on an optimal choice of noise threshold (η), visual angle (δ) and saccadic velocity threshold (v_∥ϕ,θ∥_^Thres,Saccade^). For example, with moving stimuli, the effects of noise could be augmented [10], which could compromise the ability of our algorithm to capture smooth pursuits and fixations accurately. We computed FQnS, SPQnS, misFQnS and misSPQnS as a function of the noise tolerance coefficient, ‘η’, and visual angle, δ, for the data from Experiment One. Noise tolerance coefficient, ‘η’, was varied between 0.05 and 0.4 (in steps of 0.05) and visual angle was varied between 2.5 and 4.0 (in steps of 0.5).

## Results

### Comparison of gaze event classification by the three methods

**Fig. 6:**
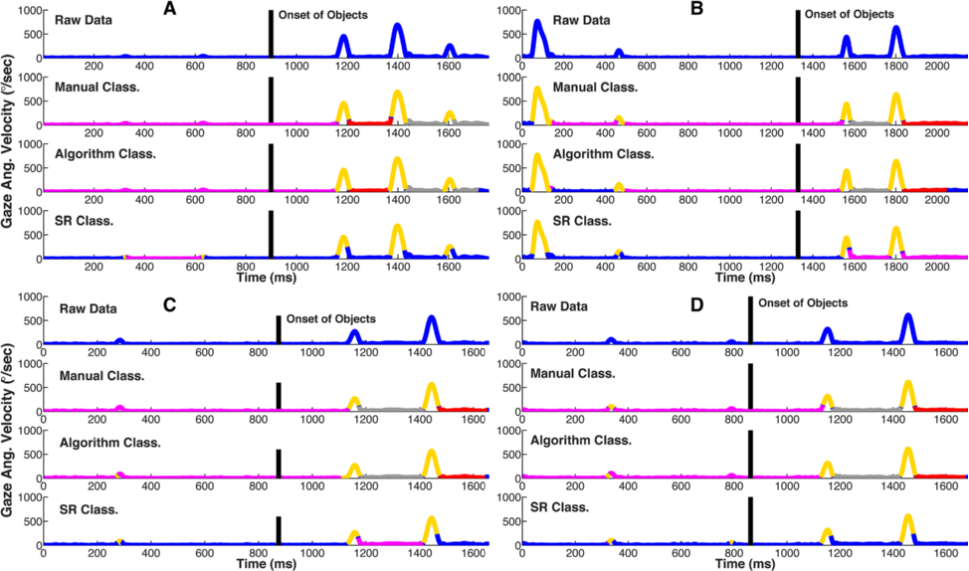
Four representative trials from Experiment One **(a-d)**, showing how the three methods classified gaze events. The gaze angular velocity (computed using Equation 6a) is plotted as a function of time. The top plot in each panel is the raw data. In the three lower plots (all four panels), we show gaze events classified by *Manual Classification*, *Algorithm Classification* and *SR Classification*. The magenta areas indicate fixation and gold indicate saccades. The smooth pursuit of the object on the left is marked in grey and the pursuit of the right object in red. The black vertical line shows the time point at which the two moving objects appeared in the workspace. The blue areas in the lower three panels indicate unclassified gaze events. All four panels (**a**, **b**, **c** and **d**) clearly show that *Algorithm Classified* gaze events were very similar to the *Manual Classified* gaze events. Furthermore, *SR Classification* did not appropriately classify fixations on the fixation circles (missing magenta areas before black vertical lines in the last plot of panels **b**, **c** and **d**)

Figure 6 shows four different trials that illustrate the main differences in how the three methods classified gaze events in Experiment One. Each trial can be separated into a *Fixation Phase* (epoch preceding object onset) and a *Movement Phase* (epoch during object movement). *Manual Classification* determined that the majority of each Fixation Phase involved fixations of the fixation circle (pink), whereas the Movement Phase included fixations (pink), saccades (gold), and pursuits (grey and blue). *Algorithm Classification* largely replicated these results, except for a section of the Fixation Phase in panel B that remained unclassified (blue). In contrast, many instances during the Fixation Phase of each trial remained unclassified by the *SR Classification* algorithm (blue). Furthermore, *SR Classification* was unable to correctly classify fixations and pursuits during the Movement Phase of each trial. Focusing closer on the saccades (gold), it is also evident that *SR Classification* incorrectly identified the offset of saccades compared to the other two methods.

Table 1 summarizes how the *Algorithm* and *SR Classifications* fared against *Manual Classification*. Note that smooth pursuits were only compared between the *Algorithm* and *Manual Classifications* because *SR Classification* does not output pursuit events. Our results show that A*lgorithm Classification* matches well with Manual Classification and far outperforms *SR Classification.* Although *SR Classification* produced fewer misclassified points than the *Algorithm Classification*, this was largely because many gaze events remained unclassified by *SR Classification* (note that unclassified points do not affect the misclassification index).

**Table 1:** Classification and misclassification rates produced by the *Algorithm* and *SR Classifications*

Variables	*Algorithm Classification*	*SR Classification*
FQnS	90.7 (70.6, 99.6)	5.6 (0, 44.1)
SQnS	92.9 (39.6, 100)	76.2 (22.4, 90.1)
SPQnS	87.7 (56.1,100)	NA
misFQnS	6.4 (0.3,28.6)	1.17 (0, 3.8)
misSQnS	4.7 (0, 30.1)	0.07 (0, 1.72)
misSPQnS	3.0 (0.5, 12.0)	NA

The gaze events computed using *Algorithm Classification* correctly classified around 90 % of fixations, saccades and pursuits. The incorrect classification rate of *Algorithm Classification* was around 5 %. These values are comparable with previously reported values for stimuli presented in vertical planes [42]. In contrast, *SR Classification* performed substantially worse at correctly identifying gaze events. Data are presented as mean (min, max)

### Classification of fixations and saccades

**Fig. 7:**
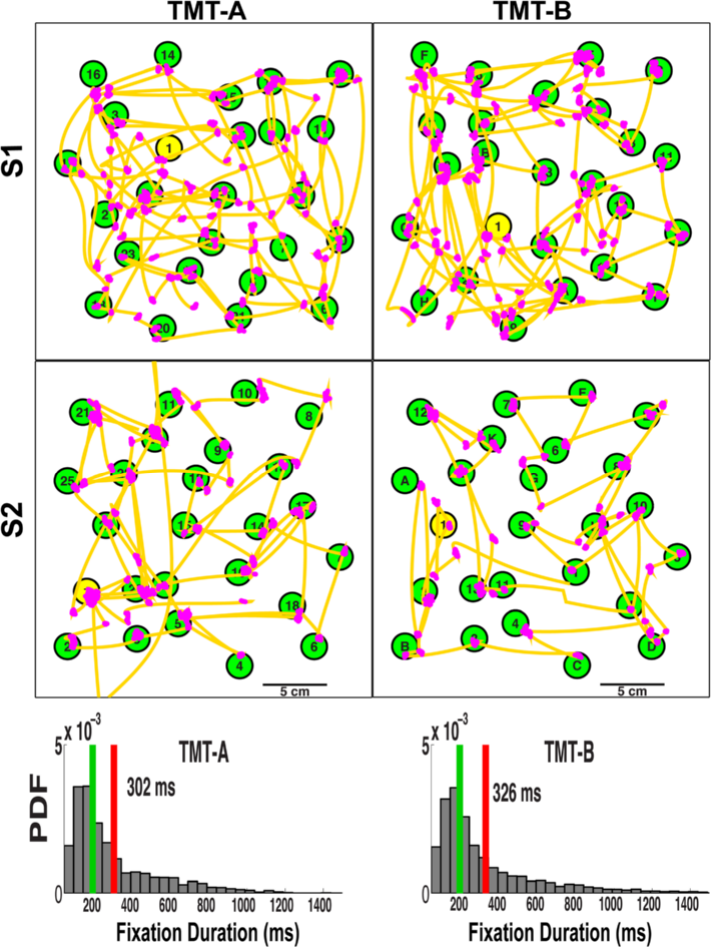
Two representative trials and probability distributions of fixation durations from Experiment Two. Top two rows: Gaze events (saccades in gold and fixations in magenta) for TMT-A (left) and TMT-B (right) tests for two representative participants (S1 and S2). The last row shows probability density functions of fixation durations for TMT-A (left bottom panel) and TMT-B (right bottom panel) performed by 37 healthy, young controls (subset of all the participants). The red vertical lines show the mean value for the two distributions (302 and 326 ms respectively) and the green lines show the medians of the two distributions (207 and 214 ms respectively)

Figure 7 illustrates the scan paths (visual search) of two exemplar subjects in Experiment Two (TMT-A, left; TMT-B, right). The scan paths show how our algorithm identified saccades (gold trajectories) used to shift gaze between targets (green numbers and letters) and fixations (magenta areas). The bottom panels of Fig. 7 show the distributions of fixation durations for all controls participants in TMT-A (left) and TMT-B (right). The vertical red and green lines show the mean and median of each distribution, respectively. The distributions displayed mean (median) fixation durations of 302 (207) ms for TMT-A and 326 (214) ms for TMT-B. These fixation durations are similar to the values (275–330 ms) previously reported for reading, visual search and scene perception tasks [43–46].

### Classification of smooth pursuits, saccades and fixations

**Fig. 8:**
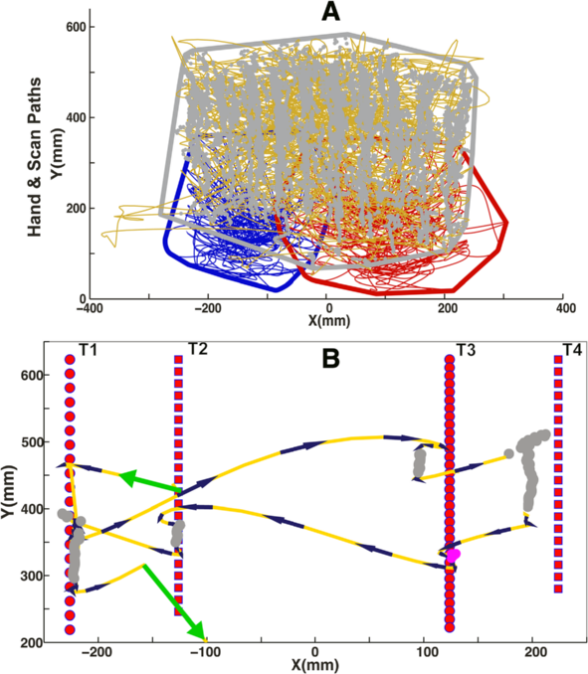
A representative trial from Experiment Three. **a** Plot shows the spatial pattern of eye and hand movements for one representative Object Hit and Avoid (OHA) trial. Hand paths show the left (blue) and right (red) hand movement during the course of an OHA trial. Eye movements are separated into saccades (gold), fixations (magenta) and smooth pursuits (grey). The polygons indicate the convex hull around the smooth pursuits (grey), right hand movement paths (red) and left hand (blue). **b** A snapshot that captures 1.8 s of smooth pursuits, fixations and saccades for the same trial as panel A for four different objects (T1, T2, T3 and T4) during the trial. The red circles (targets) and squares (distractors) show the trajectories of the four targets. The closer proximity of circles for T3 indicates that this target was traveling faster than the other three. Green arrows indicate start and end of the eye movements for the time segment. Gold lines with black arrows show saccade scan paths

Figure 8 illustrates the ability of our algorithm to classify saccades, smooth pursuits and fixations in Experiment Three (OHA task). Figure 8a shows hand movements (left, blue; right, red), saccades (gold), fixations (magenta), and smooth pursuits (grey) of a representative subject during one trial though the task (~140 s). Figure 8b shows a 1.8 s snapshot from the same trial. In this segment, the subject generated saccades (gold and black trajectories), pursuits (grey circles) and fixations (magenta circles) to orient to and foveate four objects (red circles and squares numbered T1 to T4). Gaze started at T2 (green arrow pointing left) and was followed by saccades to each of the targets, pursuits of each of the targets, and a short fixation of T3. At the end of the segment, gaze moved out of this section of the workspace (green arrow pointing right at −100, 200 mm).

### *Sensitivity of classification algorithm to Noise Threshold* (η) *and* Visual Angle *(*δ*)*

**Fig. 9:**
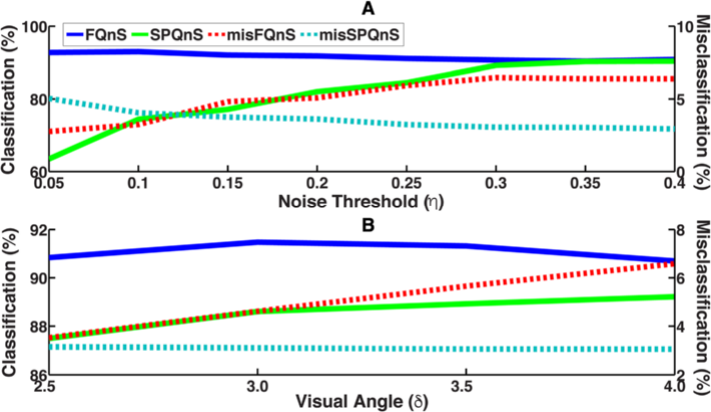
The classification (FQnS and SPQnS) and misclassification indices (misFQnS and misSPQnS) as a function of noise threshold (η, panel **a**) and visual angle (δ, panel **b**). FQnS and SPQnS are plotted on the left y-axis and misFQnS and misSPQnS are plotted on the right y-axis. For the three experiments, η = 0.2 and δ = 3.0 were used in Equations 12 and 13

Figure 9a examines the relationship between the noise threshold, ‘η’, and the accuracy of gaze event classifications. We found that fixation classification, FQnS, was relatively insensitive to ‘η’, whereas pursuit classification, SPQnS, approached an asymptote when η ≥ 0.3. We also found fixation misclassification, misFQnS, increased with increasing ‘η’ and pursuit misclassification, misSPQnS, decreased with increasing ‘η’. An optimal value of ‘η’ that maximizes FQnS and SPQnS and minimizes misFQnS and misSPQnS is between 0.1 and 0.3; we chose η = 0.2 for all the three experiments.

Figure 9b examines the relationship between the size of the foveal visual angle, ‘δ’, and the accuracy of gaze event classifications. We found that fixation classification, FQnS, was relatively insensitive to δ, whereas fixation misclassification, misFQnS, increased with δ. In contrast, pursuit classification, SPQnS, increased with δ and misclassification, misSPQnS, was relatively insensitive to δ. We chose a value of 3.0° as the optimal foveal visual angle because it maximized SPQnS and minimized misFQnS. Note that this value is slightly larger than the 2.5° reported in the literature for high visual acuity. However, direct foveation is extremely important in reading and scene perception tasks because of low visual acuity in the parafoveal and peripheral regions. In robotic environments, the visual stimuli are often more conspicuous and their shapes and color are easier to perceive. In fact, after participants have foveated a target, we have observed smooth pursuits with lags of 4.0° (data not shown).

### Saccade velocity thresholds exhibit task-dependency

**Fig. 10:**
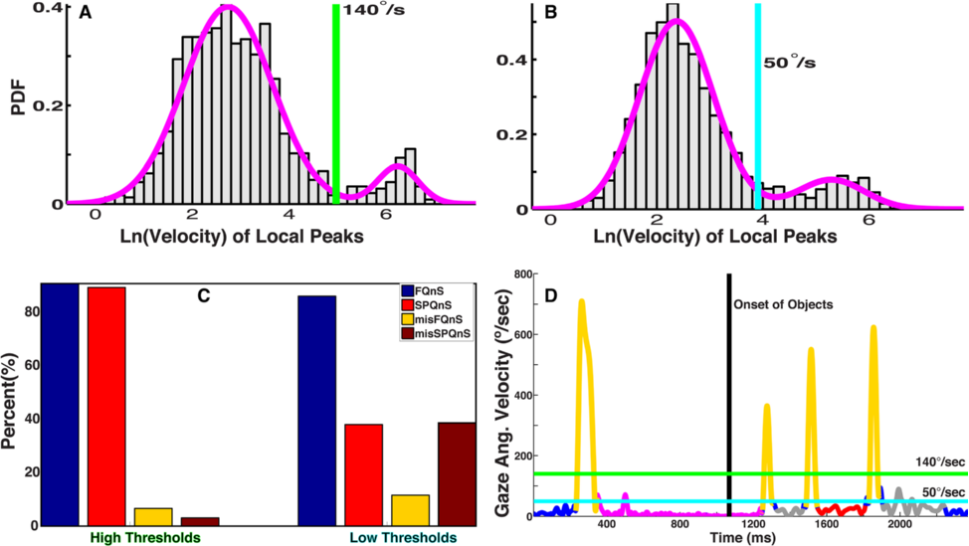
Computation of the velocity threshold for a particular experiment and participant facilitates accurate classification of gaze events. **a** Velocity threshold (140 °/s) computed using Equation 10a from all the 32 trials performed by S1 in Experiment One. This experiment involved three types of eye movements, fixations, smooth pursuits and saccades. **b** Velocity threshold (50 °/s) computed for Experiment Two (TMT-A and TMT-B) for S1. Only saccades and fixations were made in this experimental task. **c** The classification indices (FQnS and SPQnS) for Experiment One were higher and the misclassification indices (misFQnS and misSPQnS) were lower when the gaze events were classified using the high thresholds obtained from the data of Experiment One. **d** In this Figure, we show a representative trial from Experiment One. The color codes represent the same gaze events as Fig. 6. Lower velocity thresholds (cyan horizontal line) misclassify many events. A higher threshold (green horizontal line) avoids these classification errors

Figure 10 shows that the velocity threshold computed using Equation 10a is sensitive to the presence of different gaze events in a task. For example, threshold values obtained for the two participants (S1 and S2) exhibited a task-dependent shift from 140 and 111°/s in Experiment One to 50 and 20°/s in Experiment Two. Figure 10a and b show the bimodal distributions of the local velocity peaks and the computed thresholds for S1. First mode contains velocity peaks associated with noise, microsaccades and smooth pursuits in Experiment One (Fig. 10a) and only noise and microsaccades in Experiment Two (Fig. 10b). The second mode contains velocity peaks associated with saccades. This shift was expected because the stimuli in Experiment Two were stationary and only two types of eye movements were made, fixations and saccades.

These task-dependent shifts in the velocity threshold indicate that, to classify gaze events accurately, velocity thresholds should be specifically derived from the experimental data for each task. To test this, we computed the classification (FQnS and SPQnS) and misclassification indices (misFQnS and misSPQnS) for the data from Experiment One by using the velocity thresholds from Experiment One (High Thresholds) and Experiment Two (Low Thresholds). Figure 10c shows that the high thresholds (140 and 111°/s) obtained from the data in Experiment One performed much better than the lower thresholds (50 and 20°/s) obtained from Experiment Two.

To further illustrate how task-dependency of thresholds can contribute to classification and misclassification, we show a sample trial from Experiment One for S1 (Fig. 10d). The horizontal green line is the high threshold (140 °/s) and the cyan line is the low threshold (50 °/s) for S1. First, the low velocity threshold incorrectly classified smooth pursuits (second grey strip) as saccades. Second, refixations around the fixation circle (small velocity peak in magenta strip) were misclassified as saccades by the low threshold. Third, glissades at the end of saccades (short blue strip at the end of the fourth saccade) were classified as saccades by the low velocity threshold.

### Saccade velocity thresholds vary substantially across participants

**Fig. 11:**
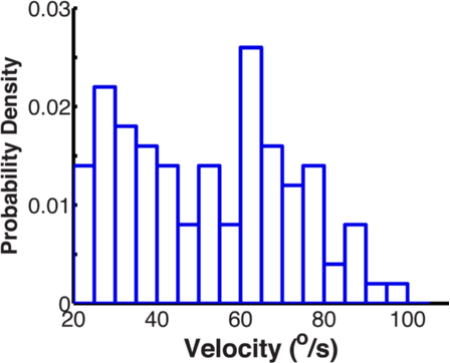
The probability distribution of velocity thresholds from 134 trials of TMT-A and TMT-B from 67 different participants (21–80 years of age, healthy and stroke survivors). The histograms show that the thresholds vary widely between individuals (range 25 and 90°/s). There were no differences between the three groups (young controls, older controls and stroke survivors)

In addition to their task-dependency, we predicted that velocity thresholds would vary across individuals. Figure 11 shows the wide range of velocity thresholds for all the 67 participants in the three groups (37 young, 16 older and 14 stroke survivors) who participated in Experiment Two (TMT-A and TMT-B). Our velocity thresholds typically ranged from 25-90°/s and were higher, on average, than the value of 30°/s proposed in the literature. Importantly, these differences between individuals indicate that computing distinct thresholds for each subject and task may improve gaze event classification. Although we also expected to see differences between groups, we did not find a significant difference between any of the three groups. This suggests that cortical lesions in chronic stroke survivors may minimally affect the velocity profile of saccades.

## Discussion

In this report we have presented a method to inverse compute ocular kinematics and classify gaze events in a robotic environment where stimuli are presented in a transverse peripersonal plane. In this section, we discuss a few features of our methods, their strengths and weaknesses, and compare them with existing methods.

### Classifying saccades, smooth pursuits and fixation in a continuous task

Previous studies have demonstrated that smooth pursuits and saccades can be accurately differentiated when a single target moves against a uniform background [47, 48]. However, in real life situations, such as driving, we rapidly switch between saccades and smooth pursuits to track multiple objects moving simultaneously [49]. To investigate this in the laboratory, we require tasks that evoke quick switching between saccades and smooth pursuits along with robust algorithms that can accurately detect and differentiate saccades and smooth pursuits.

Experiment One showed that our algorithm can classify gaze events reliably and accurately. Experiments Two and Three further demonstrated that our algorithm can separate both fixations and saccades (Experiment Two) and fixations, saccades and smooth pursuits (Experiment Three) in cognitive visuomotor tasks. In Experiment Three, we implemented a continuous visuomotor task and classified gaze events sequentially, starting with smooth pursuits, then saccades and finally fixations. Because the conditions for classifying smooth pursuits and saccades are mutually exclusive (Equations 10, 12c, 12d), the order of classification for these two gaze events does not affect classification. Fixations were classified last because of their relatively low probability of occurrence in a task where all visual stimuli are dynamic. Our method can be extended to tasks in which visual stimuli move linearly in medial-lateral or diagonal directions. For nonlinear and unpredictable movements, more complex equations are required to quantify smooth pursuits and catch-up saccades.

### Main differences between Algorithm and SR Classification

There were a few important differences between *Algorithm* and *SR Classification.* The poor performance of *SR Classification* in detecting fixations was perhaps primarily due to use of the fixed settings (0.3°, 30°/s and 8000°/s^2^). Fixations on the fixation circle were not well classified by *SR Classification* (absence of magenta curves before onset of moving objects in the last plot of Fig. 6b, c and d). We believe that this was because the fixation circle (1 cm radius) was at an approximate distance of ~60 cm from the eye and subtended a visual angle of approximately 1°. During fixations, participants’ gaze usually shifted over the entire circle area. Therefore, a fixed threshold of 0.3° of the *SR Classification* algorithm was inappropriate to quantify fixations at this distance accurately.

It is likely that the early termination of saccades with the *SR Classification* method occurs because of differences in the way that SR Research computes the gaze angular velocity. Also, smooth pursuits were often incorrectly classified as fixations by *SR Classification* (Fig. 6b and c). This was because with the given settings, *SR Classification* only categorized two gaze events, saccades and fixations, and therefore slow pursuit movements were in many instances incorrectly classified as fixations by the eye tracker. Indeed, altering the SR threshold settings could lead to better classification of events. However, the strength of our algorithm over the proprietary *SR Classification* algorithm is that it gives us direct control over defining gaze events in our programming environment for both offline calculations and online manipulation.

### Strengths and shortcomings of the proposed method

The underlying idea behind the proposed method of computing velocity thresholds to classify gaze events is inspired from the 3-component lognormal mixture model used to model fixation durations [50]. In many cognitive visuomotor studies, it has been shown that gaze velocity exhibits 1/f noise [51] and is lognormally distributed [52, 53]. In addition, gaze velocity has been shown to decrease exponentially with an increase in fixation times [54]. These results have been broadly but equivocally [for e.g. cf. 55] attributed to dynamics of eye movements in which cognition is considered to be broadly distributed over many anatomical scales and visual search is considered to be an emergent property of the entire oculomotor system [52, 56, 57]. Our proposed method uses these known features of eye movements in cognitive visuomotor paradigms to differentiate gaze events.

As an alternative to the geometric method proposed here, thresholds for gaze events could be computed in Cartesian coordinates. However, threshold values in these coordinates would have poor generalizability for stimuli located at different depths and heights in peripersonal space. Thus, it is critical to classify gaze events in the peripersonal plane using a combination of ocular kinematics and dynamics of the visual stimuli. Our method serves that purpose and creates a framework that can be extended to any eye tracking study in which visual stimuli are presented in a plane in peripersonal space.

The EyeLink 1000 used with the KINARM robot is a monocular system in which the accuracy of our method is dependent on the calibration algorithm provided by the eye tracking and robot manufacturers. The accuracy of the system in the horizontal plane is comparable to that reported by most commercially available eye tracking systems. In general, the mean gaze error reported by the system is about 0.5°. However, when the targets are far away from subjects, even a small gaze error of 0.5° can create a large absolute errors in Cartesian space (about 8–10 mm at a distance of 1 m). This should be taken into consideration when designing experimental tasks and analyzing data.

Our velocity threshold (see Equation 10a) does not parse out microsaccades [58, 59] and glissades [60]. We have not modeled these gaze events because they are beyond the scope of this study and are not commonly examined in studies of eye-hand coordination. However, including these events in our analysis is straightforward and could be implemented based on already established algorithms [21, 37].

Recent developments in algorithms for differentiating fixations and saccades have provided another novel approach to classify saccades and fixations [61]. The algorithm proposed by König and Buffalo does not rely on thresholds (we used Equations 10 and 13). Their algorithm provides an alternative method based on state space models that can be combined with the geometric component of our model to provide reliable classification of saccades and fixations in robotic environments.

### Comparison with other methods for measuring spatial gaze

Others have proposed methods for computing gaze point-of-regard (POR) in 3D space in head-restrained conditions [62, 63]. Recent methodological developments have also allowed measurement of gaze POR in space when the head is unrestrained [64–66]. This method requires a binocular eye tracking system and combines measures of eye-in-head (from a video-based gaze-tracker) and head-in-space (from a motion capture system) to provide a vergence based POR in 3D space. The calibration routines for these systems are computationally intensive and require an extensive amount of time to map out the entire 3D space. Although these systems have been widely used in immersive environments for studying problems of navigation in space [reviewed in 10], our the ability to rapidly calibrate individuals with remote monocular systems makes them much more feasible for studies of older adults, children and patient populations.

## Conclusions

Our proposed method provides a unique approach for using monocular remote eye tracking to compute ocular kinematics from Cartesian POR data in the transverse peripersonal plane. In this method, we first inverse compute the ocular kinematics by transforming the Cartesian gaze POR data to spherical coordinates. After obtaining ocular kinematics, we compute gaze velocity thresholds that are task, environment and individual specific. Finally, we combine ocular kinematics, velocity thresholds and kinematics of the visual stimuli to accurately classify gaze events (saccades, fixations, smooth pursuits). The main advantage of this method is that it provides direct control to the experimenter for robust offline analyses of gaze events and online experimental control.

## Additional files

Additional file 1:
**Is a .png file that shows a visual representation of the computation involved in Equation 10a.** (PNG 407 kb) Additional file 2:
**Is a .m MATLAB function that contains subroutines to implement Equations 2–10.** (M 29 kb) Additional file 3:
**Is a .mp4 file that contains videos of trials of all three experiments.** (MP4 9645 kb)

## Abbreviations

POR: Point of regard
TMT: Trail making test
OHA: Object hit and avoid
FVR: Foveal visual radius
FVC: Foveal visual circle
