# Supplementary figures and images for: A geometric method for computing ocular kinematics and classifying gaze events using monocular remote eye tracking in a robotic environment
Source: J Neuroeng Rehabil. 2016 Jan 26;13:10. doi: 10.1186/s12984-015-0107-4 (PMC4728792; doi:10.1186/s12984-015-0107-4)

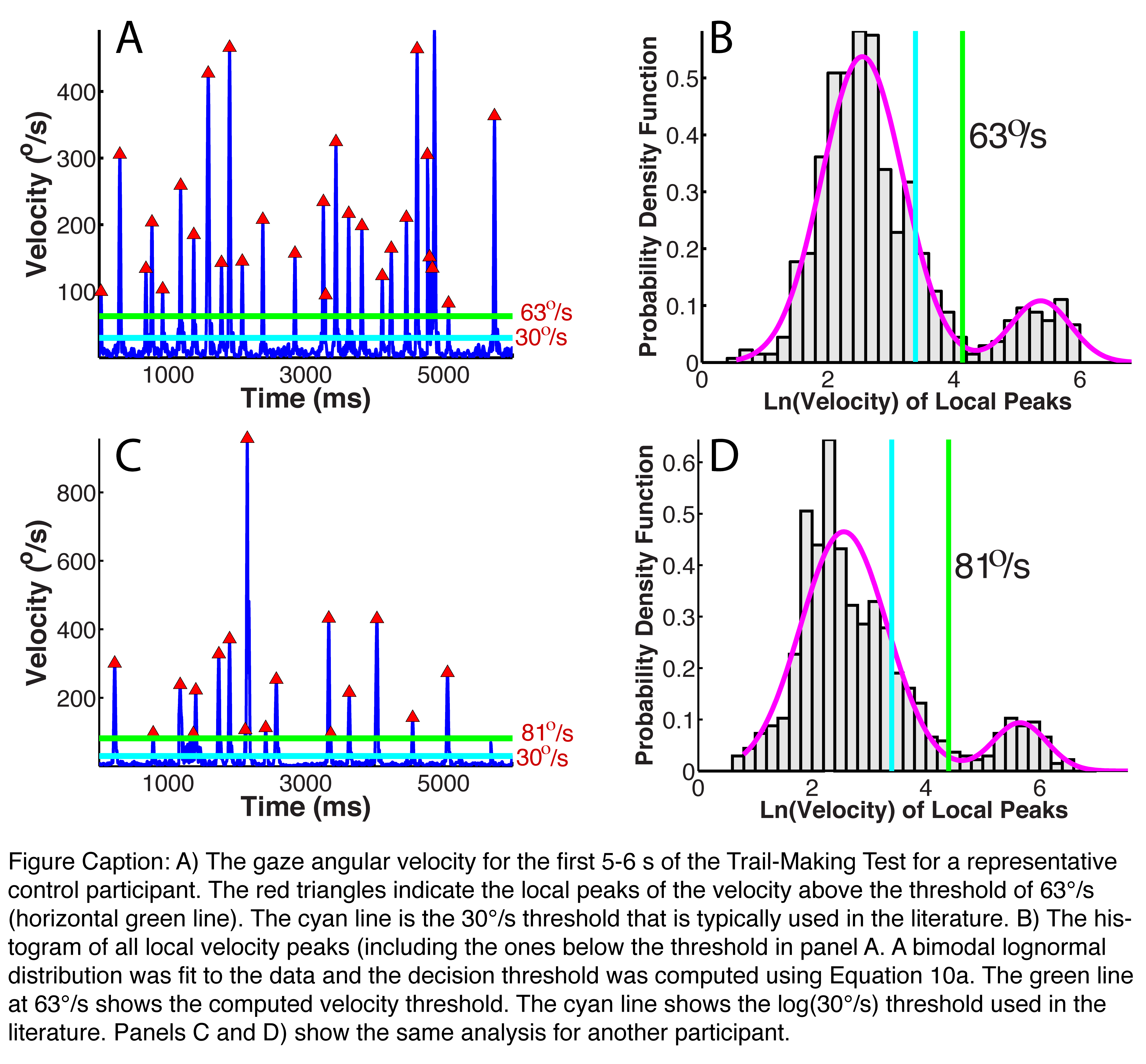

Supplement: Additional file 1: — Is a .png file that shows a visual representation of the computation involved in Equation 10a. (PNG 407 kb) [file 12984_2015_107_MOESM1_ESM.png]
